# Evaluation of a Model (RUMINANT) for Prediction of DMI and CH_4_ from Tropical Beef Cattle

**DOI:** 10.3390/ani13040721

**Published:** 2023-02-17

**Authors:** Alejandro Ruden, Bernardo Rivera, Julio Ernesto Vargas, Secundino López, Xiomara Gaviria, Ngonidzashe Chirinda, Jacobo Arango

**Affiliations:** 1 Alliance Bioversity International (CIAT), Palmira 763537, Colombia; 2 Departamento de Producción Agropecuaria, Universidad de Caldas, Manizales 170001, Colombia; 3 Instituto de Ganadería de Montaña, Departamento de Producción Animal, Universidad de León, 24007 León, Spain; 4 Agricultural Innovation and Technology Transfer (AITTC), Mohammed VI Polytechnic University (UM6P), Ben Guerir 43150, Morocco

**Keywords:** fermentation, mathematical model, greenhouse gases, methane, RUMINANT

## Abstract

**Simple Summary:**

Methane (CH_4_) is a byproduct of the digestion of cattle; this gas has a greenhouse effect in the atmosphere, which contributes to global warming. As the direct measurement of methane demands time and resources, the objective of this work was to evaluate the ability of a mathematical model (RUMINANT) to predict methane emissions from livestock. With this objective, methane measurements were made in individual chambers, and the results were compared with methane emissions estimated by the RUMINANT model. The model showed a high capacity to predict dry matter intake. However, in the case of methane emissions, it did not. The model substantially underestimates methane emission in all diets (six) but one including *Leucaena diversifolia.* On diets without Leucaena, the precision of the model was adequate, but on diets with Leucaena, there was not a linear regression between the observed and simulated methane emission values. This may be an effect of the anti-methanogenic factors of Leucaena that are not accounted for by the RUMINANT model. This study contributes to improving national inventories of greenhouse gases from the livestock of tropical countries.

**Abstract:**

Simulation models represent a low-cost approach to evaluating agricultural systems. In the current study, the precision and accuracy of the RUMINANT model to predict dry matter intake (DMI) and methane emissions from beef cattle fed tropical diets (characteristic of Colombia) was assessed. Feed intake (DMI) and methane emissions were measured in Brahman steers housed in polytunnels and fed six forage diets. In addition, DMI and methane emissions were predicted by the RUMINANT model. The model’s predictive capability was measured on the basis of precision: coefficients of variation (CV%) and determination (R^2^, percentage of variance accounted for by the model), and model efficiency (ME) and accuracy: the simulated/observed ratio (S/O ratio) and slope and mean bias (MB%). In addition, combined measurements of accuracy and precision were carried out by means of mean square prediction error (MSPE) and correlation correspondence coefficient (CCC) and their components. The predictive capability of the RUMINANT model to simulate DMI resulted as valuable for mean S/O ratio (1.07), MB% (2.23%), CV% (17%), R^2^ (0.886), ME (0.809), CCC (0.869). However, for methane emission simulations, the model substantially underestimated methane emissions (mean S/O ratio = 0.697, MB% = −30.5%), and ME and CCC were −0.431 and 0.485, respectively. In addition, a subset of data corresponding to diets with Leucaena was not observed to have a linear relationship between the observed and simulated values. It is suggested that this may be related to anti-methanogenic factors characteristic of Leucaena, which were not accounted for by the model. This study contributes to improving national inventories of greenhouse gases from the livestock of tropical countries.

## 1. Introduction

In light of growing concern about climate change, due to the contribution of livestock to greenhouse gas inventories and the difficulty of in-field emission measurements for diverse physiological stages and forage diets [1], it has become desirable to use simulation models to estimate bovine enteric methane emission. These models reduce the time and cost associated with research, allowing the experimenters to examine responses to changes in farming conditions [2]. Ruminal methane emissions can be estimated from data on dry matter intake (DMI) through statistical models [3,4]; however, when they are applied outside of original conditions, serious uncertainties appear [5]. Dynamic mechanistic models based on mathematical descriptions and the biochemistry of ruminal fermentation are also used to estimate these emissions [6].

In the case of Colombia, characteristic of tropical conditions, a deterministic model (RUMINANT) has been the basis for estimating national methane emissions from its bovine livestock systems and for guiding the corresponding National Appropriate Mitigation Actions (NAMAs) [7]. The model has also been used to estimate methane emissions from Africa [8] and global bovine livestock systems [9]. Apart from methane, the model also estimates dry matter intake, manure, and nitrogen excretion, as well as meat and milk production [10,11]. It uses baseline data that describe the animal and the quality of the pastures to evaluate the effect on methane emissions. The predictive capacity of the DMI of the RUMINANT model has been validated using databases, including a wide range of diet qualities from temperate and tropical countries [9,12]. However, there has been no validation of the RUMINANT model regarding methane emission under the typical feeding characteristics of the lowlands of Tropical America. Validation is addressed based on two descriptors: (i) precision or dispersion between the output values of the model (simulated) and the values measured experimentally (observed), and (ii) accuracy or mean distance between the simulated values and observed values [13,14]. Because validation quantifies the predictive capacity of a model, it is expected to improve future national communications of methane emissions.

The objective of this research was to evaluate the predictive capacity of the RUMINANT model to estimate voluntary DMI and enteric methane emissions from beef cattle fed tropical forage diets used in Colombia.

## 2. Materials and Methods

### 2.1. Area of Study

The research was conducted at the International Center for Tropical Agriculture, CIAT (Valle del Cauca), which is located at 3°30′ north latitude, 76°21′ west longitude, at 1008 m.a.s.l., with an average temperature of 23.8 °C, annual precipitation of 938 mm, and relative humidity of 76%.

### 2.2. Experimental Design

The study was planned as a correlational research design, analyzing the relationship between the observed and predicted values of DMI and methane production from grazing cattle. To approach the objective, six tropical pasture diets with a wide range of chemical and botanical compositions were used (Table 1), with four replicates (steers) per diet. The diets were evaluated over six consecutive experimental periods.

### 2.3. Forage Diets

Steers were fed a series of six forage diets:

1. Toledo grass (*Brachiaria brizantha* cv. CIAT 26110);
2. Cayman grass (*Brachiaria hybrid cv. CIAT BR 02/1752*);
3. Star grass (*Cynodon plectostachius*) and tropical kudzu (*Pueraria phaseoloides*) in a 70:30 ratio;
4. Cayman grass and Leucaena (*Leucaena diversifolia)* in a 70:30 ratio;
5. Cayman grass and Leucaena (*Leucaena leucocephala)* in a 70:30 ratio;
6. Toledo grass, Leucaena (*L. diversifolia)*, and Canavalia (*Canavalia brasiliensis*) in a 70:15:15 ratio.

Nutritional quality of the forage diets is shown in Table 1. The neutral detergent fiber (NDF), crude protein (CP), and ether extract (EE) were determined through the methodologies described by Van Soest et al. [15], Bradstreet [16], and Horwitz et al. [17]. In vitro dry matter digestibility (IVDMD) was determined by the technique of Tilley and Terry [18].

### 2.4. Animals

A herd of 20 Brahman steers (ca. 20 months of age and average body weight 206 +/− 36 kg) grazed consecutively in each of the 6 pastures described in Table 1. The steers were kept in each pasture over an adaptation period of 20 days. At the end of each period, four steers were randomly selected to be individually confined in a polytunnel housing facility. In the individual stalls, the steers were fed fresh grass herbage harvested daily, cut from the same sward grazed before confinement. During a measurement period of six days, DMI intake and methane emissions for each animal were determined and recorded daily. After the measurement period, the herd was regrouped, and all 20 steers were moved to a new grazing plot (with a different pasture composition) initiating a new adaptation grazing period for the evaluation of that particular forage diet. This process was repeated over six experimental periods to complete the evaluation of the six forage diets (Table 1).

At the beginning of the experiment, the animals underwent a veterinary inspection, as well as internal and external parasite treatments.

### 2.5. Determination of Intake

DMI (kg/animal/day) was obtained by measuring the difference between the amount of feed provided and the amount of feed rejected during the time the animals were kept in the polytunnel. The confined animals received the same forage diet they had been grazing, and they also had access to mineral salt and water on demand. The Leucaena was collected manually, simulating the harvest height and the bite size of the animals in the silvopastoral system. Thus, mainly leaves and some thin stems (<0.5 cm) were collected. Samples of all dietary components of the feed provided were taken for quality evaluation.

### 2.6. Gas Measurement

Methane production (L/animal/day) was measured using the polytunnel technique described by Lockyer [19]. The polyethylene chamber was divided into four independent compartments, each with the capacity to house one animal and equipped with hermetic sealing, an internal fan to homogenize the air, and an exhaust fan for sample taking.

As mentioned above, after each 20-day period of adjustment to the forage diet, 4 out of the 20 animals were housed inside the polytunnel for 6 days. During the first five days, the animals adapted to the facilities and management. On the sixth day, samples were taken from the air contained in the chambers every 60 min for 24 h by means of an exhaust fan that was set at an extraction rate of 0.9 m^3^ s^−1^. Every gas measurement consisted of the collection of gas samples every 20 s. The methane concentration was determined by a portable infrared spectrometer (Gasmet™) [20,21] that was connected to the output of the extractor fan. The equipment was calibrated with ultrapure dinitrogen gas grade 5.0 according to manufacturer instructions. At the end of each measurement, the polytunnel doors were opened to allow for ventilation and to equalize methane concentration with the ambient air.

### 2.7. Simulation of Intake and Methane Emissions

The fermentation process and end-products were simulated for each animal by applying the widely used RUMINANT model designed by Herrero et al. [10]. Regarding the compositional quality of the forages, crude protein, neutral detergent fiber, fat, and ashes were entered into this model, as well as the non-structural carbohydrate (NSC) content (NSC = OM% − CP% − NDF% − EE%) and IVDMD. NSC and IVDMD were used to calculate the carbohydrate fractions as defined in the up-cited original RUMINANT model. Other inputs were the characteristics of the randomly selected animal: sex (all male), current weight and potential weight, along with its activity (housed indoors), and feeding system (stall-fed). With this information, the model generated simulated values for voluntary intake (kg DM/day) and methane emission (L/animal/day).

### 2.8. Predictive Capability of the RUMINANT Model

The predictive capacity of the model was determined by evaluating its accuracy and precision using the observed values within the polytunnel (observed values, O) and the corresponding prediction (simulated values, S).

The accuracy of the model was established through (i) the S/O ratio of individual data; (ii) the slope of the linear regression with a zero intercept between “O” and “S” [13]; and (iii) the mean bias (MB) or mean difference between the observed and simulated values. Regarding the S/O ratio, a value of one (1) corresponds to the maximum accuracy of the model; lower or higher values would mean some degree of underestimation or overestimation, respectively, of a particular variable (methane emissions or dry matter intake). In the case of the slope, a value of one (1) corresponds to maximum accuracy. In the case of the mean bias, the lower the absolute value, the more accurate the model [14]; with negative values indicating that the simulation underestimates the observed values (Table 2).

Precision was measured through (i) the coefficient of variation (CV, %) of the data set; (ii) the coefficient of determination (R^2^) of the linear regression; and (iii) the proportion of variance explained by the line Y = f(X1,…,Xp), which is considered one of the best descriptors for precision and is known as model efficiency (ME) [22]. The coefficient of variation is inversely proportional to precision. On the other hand, the coefficient of determination (R^2^) is directly proportional to precision, as it indicates the proportion of variation in the values explained by the model [23]. With model efficiency (ME), one (1) is the result of highest precision (Table 2).

Combined measurements of accuracy and precision were also performed by means of (i) the mean square prediction error (MSPE) [24] and (ii) the concordance correlation coefficient (CCC) [25] (Table 2).

### 2.9. Sensitivity Analysis

For the sensitivity analysis of the model, 256 simulations were performed using the information obtained in the laboratory about the compositional quality for each of the forage diets under study: CP, NDF, ashes, and NSC, as well as IVDMD. The simulations were performed by entering the nutritional quality values of each forage base into the model, except one, whose value was sequentially replaced by the minimum and maximum values recorded in the database. This was performed until the values of all nutritional components of all forage diets had been substituted. The information thus generated underwent principal component analysis using R statistical program [26,27] to compare the modified variables with the ones obtained with the model and, thus, establish the influence and statistical significance of each nutritional component on simulated methane production.

## 3. Results

### 3.1. Accuracy and Precision of DMI Simulation

**Figure 1:**
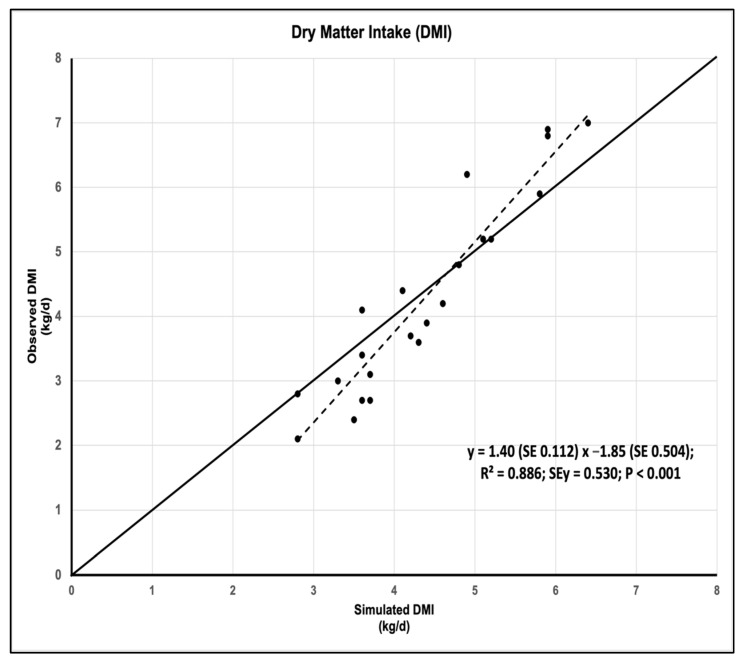
Relationship between the simulated and observed DMI.

The accuracy measurements of the RUMINANT model for simulating DMI are presented in Table 3. The average S/O ratio (1.07) and mean bias (2.23%) indicate that the model accurately predicts DMI. However, the slope of linear regression was relatively high (1.4). Figure 1 shows how the model both slightly overestimates and underestimates DMI.

The coefficient of variation of the values of the S/O ratio (CV = 17.0%), the coefficient of determination of the linear regression of the simulated DMI against the observed one (R^2^ = 0.886), and the efficiency of the model (ME = 0.809) show that the model precisely simulates the DMI (Table 3).

Moreover, the analysis of MSPE for the relationship between the observed and simulated values showed that the main component (59.8% of MSPE) was the error due to random data disturbance. There were no significant differences (*p* = 0.505) between the observed and simulated values, and therefore, error attributed to the overall bias of prediction accounted for only 2.1% of MSPE. In contrast, the error attributed to the deviation in the regression slope from unity was 38% of MSPE, as, in fact, low intakes (less than 4.6 kg/d) were overestimated, and high intakes (more than 4.6 kg/d) were underestimated with the RUMINANT model (Figure 1). The concordance correlation coefficient (CCC = 0.87) showed high reproducibility of the actual intakes using this model, with both precision and accuracy coefficient approaching unity (Table 3).

In general terms, the RUMINANT model has high accuracy and high precision in simulating the DMI of Zebu animals fed tropical forage diets.

### 3.2. Simulation of Methane Emissions

**Figure 2:**
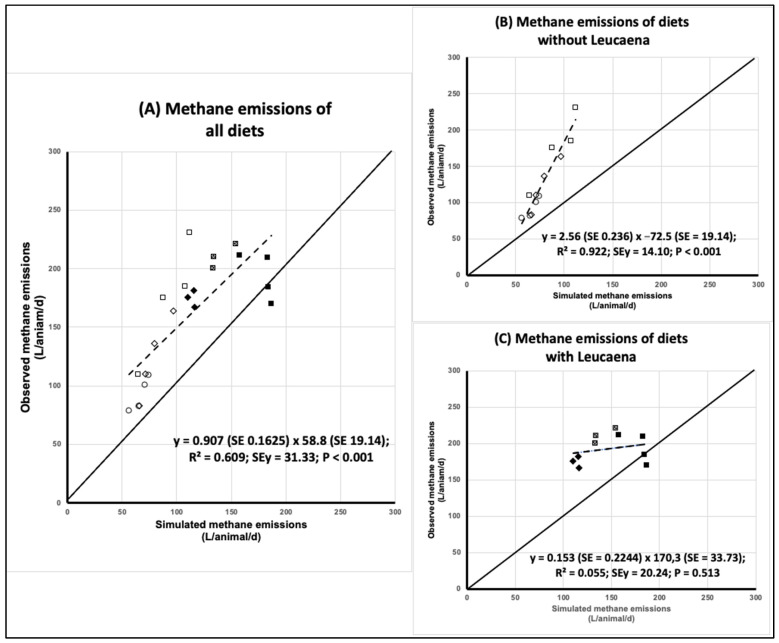
Relationship between the simulated and observed methane emissions for (**A**) all diets; (**B**) diets without Leucaena: ◇ Toledo grass, ◻ = Cayman grass, and ◯ = Star grass plus kudzu (70:30); and (**C**) diets with Leucaena: ◼ = Cayman grass plus *L. diversifolia* (70:30), ▩ = Cayman grass plus *L. leucocephala* (70:30), and ◆ = Toledo grass plus Canavalia plus *L. diversifolia* (70:15:15).

Unlike the DMI simulation, methane emission was substantially underestimated by the model in all but two of the data (graph “a” of Figure 2). It was also noticed that (i) points were not evenly distributed along the fitted regression line; (ii) data that exhibited an overestimation of the simulated methane emission pertain to diets with *Leucaena diversifolia* at 30% of DM; and (iii) the group of points corresponding to diets with Leucaena did not particularly suit the line fitted by the regression. Thus, the evaluation of accuracy and precision was also carried out for two subsets of data: diets with and without Leucaena (Table 4). Separate regression analysis indeed showed that in diets without Leucaena, the simulated and observed data of methane emission followed a linear relationship, while those with Leucaena did not (graphs “b” and “c” of Figure 2).

#### 3.2.1. Accuracy

The average S/O ratio (0.697) demonstrated a noticeable underestimation of methane emissions as simulated by RUMINANT. However, the underestimation appeared to be greater in diets without (S/O = 0.637) than with (S/O = 0.769) Leucaena. While in diets without Leucaena, the slope was high (2.559), and in diets with Leucaena, the slope was low (0.153) and not different from zero (Figure 2). Mean bias was also higher without than with Leucaena. As a percentage of average observed emissions, it was −39.2 % and −23.5% for forage basis without and with Leucaena, respectively. The magnitudes of the underestimations assessed by MB and S/O ratio were close to each other (Table 4). Thus, under tropical conditions, the RUMINANT model substantially underestimates the methane production of Zebu animals consuming forage diets with and without Leucaena.

#### 3.2.2. Precision

As measured by the coefficient of variation of the S/O ratio, the precision of the RUMINANT model in simulating methane emissions was considered reasonable; the CV was 15.6% and 21.7% for diets without and with Leucaena, respectively. As measured by the coefficient of determination, in diets without Leucaena, precision was outstanding (R^2^ = 0.922), and it was poor in diets with Leucaena (0.055). However, ME shows that precision was inferior in diets without Leucaena (ME = −0.659), while in diets with Leucaena, ME (−7.594) reflects the lack of a linear relationship between the observed and simulated methane emissions. Thus, in the case of the forage diets with Leucaena, simply using the average value of observed emissions is more precise than the simulated values produced by the RUMINANT model (Table 4 and Figure 2).

#### 3.2.3. Combined Accuracy and Precision

With regards to the data set of all forage diets, the overall bias was 72%, with significant (*p* < 0.001) differences between the observed (mean value, 159 L/animal/day) and simulated (mean value, 110 L/animal/day) methane production. Deviation was a minor component (0.4% of total MSPE), as the slope was not significantly different from unity. The concordance coefficient showed acceptable precision and accuracy resulting in a medium and limited reproducibility (Table 4).

A direct comparison between forage diets with or without Leucaena in terms of the absolute values of MSPE is of little meaning because these were calculated on different datasets. However, in both sub-datasets, most of the MSPE had to be attributed to bias between the observed and simulated values (between 69 and 75%), as the RUMINANT model underestimated methane production in all cases. The partition analysis also showed that in both separate datasets, the error due to slope accounted for approximately 20% of the MSPE. However, the slope was significantly greater than 1 in diets without Leucaena, while in diets with Leucaena, it was lower than 1 and not significantly different from zero. The concordance between the observed and simulated values was low for both separate datasets due to low accuracy with diets without Leucaena (despite high precision) and low accuracy and precision in the dataset of diets with Leucaena.

### 3.3. Sensitivity Analysis of the RUMINANT Model to Forage Quality Values

The sensitivity analysis showed that the contents of NDF, NSC, EE, and ashes of forages have an inverse relationship with methane emissions (Table 5). This means that an increase in these input values reduces the value of methane production simulated by the RUMINANT model. On the contrary, crude protein and IVDMD have a direct relationship with the amount of methane simulated by the model.

## 4. Discussion

The RUMINANT model showed a valuable predictive capability, in terms of precision and accuracy, to simulate DMI by Zebu steers with both forage diets included in the study. The model is suitable for calculating a DMI estimate, which, many times, is lacking and hinders the analysis of agricultural systems [28,29]. This is particularly important because DMI is a crucial factor for an accurate estimation of bovine enteric methane emissions [3,4,5].

On the other hand, the predictive performance of the RUMINANT model to simulate methane production was much poorer than in the case of DMI. It is worth mentioning that the model had extremely low precision on forage diets with Leucaena compared to diets without Leucaena. The presence of Leucaena had an influence on methane emissions that were not simulated by the model. This influence seemed particularly strong in the case of *L. diversifolia* fed at 30% of DM. In fact, it has been reported that whereas *L. diversifolia* reduced methane production, *L. leucocephala* did not [30]. Nonetheless, in other studies, the addition of *L. leucocephala* to a diet of Star grass increased DMI and productivity without increasing methane emission [31,32]. However, it is known that the effect of *L. leucocephala* on methane reduction depends on the level of incorporation in the diet, and the optimum level has been set at between 20–40% of ration DM [33]. As for accuracy, it was found that the RUMINANT model substantially underestimated methane emission in both cases (without and with Leucaena), although the underestimation was slightly greater in diets without Leucaena. The simulation of methane emission is based on fermentation stoichiometries [8] and does not consider the determinant role of the archaeal population [34], the complex interactions between the substrates fermented and the major types of micro-organisms [35], and the presence of condensed tannins in Leucaena that are known to reduce methane emission [36].

Even though sensitivity analysis did not account for links between nutritional components of the feed, the results were as expected. NDF, NSC, EE, and Ashes have an inverse relation to methane emissions. Previous research found that NDF was the most sensitive variable limiting DMI on the RUMINANT model [9] from which a reduction in the emission of methane would be expected [37]. However, at fixed DMI, an increase in the content of NSC is known to lean fermentation outputs towards the production of propionic acid at the expense of acetic acid and methane [38]. EE in the diet can have an inverse relationship to methane emission because fatty acids of galactolipids are not fermented in the rumen, only sugars are [39,40] or may directly interfere with methanogenesis [37]. An inverse relationship between CP content or IVDMD and methane could be also expected. Increasing CP content above limiting levels (<8%) allows for overcoming the constraint of bacterial growth and fermentation [41,42]. An increase in IVDMD means a greater extent of fermentation and more availability of structural carbohydrates for fermentation, resulting in increased production of acetate and methane. [43].

Considering that feed for beef production systems in Colombia does not normally include Leucaena, it seems valuable to adjust the RUMINANT simulated methane emissions by using the corresponding mean bias of the model (MB = 39.2%), as suggested by [44]. In addition, due to the rapid growth in the adoption of silvopastoral systems [45], it would be worthy to consider adjusting the model algorithms to incorporate anti-methanogenic properties of some secondary metabolites of trees, shrubs, and leguminous species [46]. Additionally, to improve the quality of national bovine methane emission inventories, it is worth considering the evaluation of simple models based on DMI that can be well established using the RUMINANT model.

## 5. Conclusions

The RUMINANT model is a highly powerful tool (accuracy and precision) to simulate the DMI of Brahman Zebu steers fed tropical forage diets characteristic of Valle del Cauca (Colombia), with and without Leucaena. On the basis of accurate information on animals and diet composition, simulated DMI is useful to support decision-making in research, technology transfer, and development processes in cattle-fattening systems. For diets without Leucaena, the model simulates methane emissions with reasonable precision. However, adjustments are needed to improve the accuracy of this simulation because emissions are underestimated by 39.2%. When forage diets included Leucaena, there was not a linear relationship between the observed and simulated methane emission values. These results were possibly related to anti-methanogenic compounds in Leucaena, and it may be valuable to incorporate algorithms that take this effect into account. The sensitivity analysis showed that the model responds in an expected way to changes in the nutrient content of the forage base. There was an inverse relationship between methane production and NDF content, NSC, ashes, and fat, while there was a direct relationship with IVDMD and CP, which seems to recognize situations where their reduced availability restricts fermentation.

## Figures and Tables

**Table 1:** Nutritional qualities of the forage diets used (as % of DM).

Forage Diets	CP (%)	NDF (%)	NSC (%)	EE (%)	Ashes (%)	IVDMD (%)
Toledo Grass ^1^	6.5	69.2	11.4	2.51	10.5	64.1
Cayman Grass ^2^	8.3	68.2	8.8	2.51	12.1	61.3
Star Grass ^3^ plus Kudzu ^4^ (70:30)	11.2	72.9	3.4	2.97	9.6	58.7
Cayman Grass ^2^ plus Leucaena ^5^ (70:30)	14.7	56.8	12.5	3.46	12.5	60.4
Cayman Grass ^2^ plus Leucaena ^6^ (70:30)	11.0	62.5	10.9	3.78	11.9	61.6
Toledo Grass ^1^ plus Canavalia ^7^ plus Leucaena ^5^ (70:15:15)	10.8	66.5	10.2	2.92	9.6	61.2

CP—crude protein; NDF—neutral detergent fiber; NSC—non-structural carbohydrate; EE—ether extract; and IVDMD—in vitro dry matter digestibility. ^1^ *Brachiaria brizantha*; ^2^ *Brachiaria hybrid*; ^3^ *Cynodon plectostachius*; ^4^ *Pueraria phaseoloides*; ^5^ *Leucaena diversifolia*; ^6^ *Leucaena leucocephala*; and ^7^ *Canavalia brasiliensis*.

**Table 2:** Model accuracy and precision descriptors.

	Equation
Accuracy	
Mean of S/O ratio $\left(M\left(\frac{S}{O}\right)R\right)$	$M\left(\frac{S}{O}\right)R=\frac{\sum \left(\frac{S}{O}\right)}{n}\;$
Slope of the linear regression, *β*_1_	$\beta_{1}=\frac{cov_{os}}{SD_{s}^{2}}=r\frac{SD_{o}}{SD_{s}}$
Mean bias, MB (%)	$MB=\frac{\;\left(\frac{\sum_{i=1}^{n}\left(O_{i}-S_{i}\right)}{n}\right)}{\bar{O}}\times 100$
Precision	
Coefficient of variation of S/O (%)	$CV=\frac{SD\;\left(\frac{S}{O}\right)}{\bar{\left(\frac{S}{O}\right)}}\times 100$
Coefficient of determination, R^2^	$R^{2}=1-\frac{{\left(\sum \left(S_{i}-\bar{S}\right)\left(O_{i}-\bar{O}\right)\right)}^{2}}{\left(\sum {\left(S_{i}-\bar{S}\right)}^{2}\right)(\sum {\left(O_{i}-\bar{O}\right)}^{2})}$
Model efficiency, ME	$ME=1-\frac{\sum {\left(S_{i}-O_{i}\right)}^{2}}{\sum {\left(O_{i}-\bar{O}\right)}^{2}}$
Combination of accuracy and precision	
Mean square prediction error, MSPE	$MSPE=\frac{1}{n}\overset{n}{\underset{i=1}{\sum }}{\left(S_{i}-O_{i}\right)}^{2}$
Bias, B (%)	$B=\frac{\;{\left(\bar{S}-\bar{O}\right)}^{2}}{MSPE}\times 100$
Slope, Sl (%)	$Sl=\frac{\;SD_{s}^{2}{\left(1-\beta_{1}\right)}^{2}}{MSPE}\times 100$
Random, Rd (%)	$Rd=\frac{\;\left(1+r^{2}\right)SD_{o}^{2}}{MSPE}\times 100$
Concordance correlation coefficient, CCC	$CCC=\frac{2cov_{os}}{SD_{o}^{2}+SD_{s}^{2}+{\left(\bar{S}-\bar{O}\right)}^{2}}$
Accuracy component, Ca	$Ca=\frac{2SD_{o}SD_{s}}{SD_{o}^{2}+SD_{s}^{2}+{\left(\bar{S}-\bar{O}\right)}^{2}}$
Precision component (Pearson correlation coefficient), R	$R=\frac{cov_{os}}{SD_{o}SD_{s}}$

O—observed value; S—simulated value; ß1—slope; ε—error; SD—standard deviation; and cov—covariance.

**Table 3:** Descriptors of accuracy and precision of the RUMINANT model to simulate dry matter intake (DMI) on forage diets without and with Leucaena.

	Without Leucaena
N	22 ^1^
Accuracy	
Mean S/O ratio	1.07
Slope	1.40
Mean bias (%)	2.23
Precision	
Coefficient of variation of S/O ratio (%)	17.0
R^2^	0.886
Model efficiency	0.809
Combined accuracy and precision	
Mean square prediction error	0.426
Bias (%)	2.1
Slope (%)	38.0
Random (%)	59.8
Concordance correlation coefficient	0.869
Ca	0.923
R	0.941

^1^ Two animals were excluded because they presented health alterations not related to diet.

**Table 4:** Descriptors of accuracy and precision of the RUMINANT model in simulating methane emissions for diets of tropical forages.

	All Diets	Diets without Leucaena	Diets with Leucaena
N	22	12	10 ^1^
Accuracy			
Mean S/O ratio	0.697	0.637	0.769
Slope	0.907	2.559	0.153
Mean bias (%)	−30.5	−39.2	−23.5
Precision			
Coefficient of variation of S/O ratio (%)	21.1	15.6	21.7
R^2^	0.609	0.922	0.055
Model efficiency	−0.431	−0.659	−7.594
Combined accuracy and precision			
Mean square prediction error	3259	3500	2969
Bias (%)	72.2	74.6	69.3
Slope (%)	0.4	20.7	19.7
Random (%)	27.4	4.7	11.0
Concordance correlation coefficient	0.485	0.303	0.078
Ca	0.621	0.316	0.330
R	0.780	0.960	0.235

^1^ Two animals were excluded because they presented health alterations not related to diet.

**Table 5:** Influence of the variables entered in the RUMINANT model on methane emissions.

Variable	Effect on Methane Emissions
NDF	-
Non-structural carbohydrates	-
Fat	-
Ashes	-
Crude protein	+
IVDMD	+

## Data Availability

The data presented in this study are available on request from the corresponding author.
